# Experimental measurements and CFD modelling of hydroxyapatite scaffolds in perfusion bioreactors for bone regeneration

**DOI:** 10.1093/rb/rbad002

**Published:** 2023-01-23

**Authors:** Alessandro d’Adamo, Elisabetta Salerno, Giuseppe Corda, Claudio Ongaro, Barbara Zardin, Andrea Ruffini, Giulia Orlandi, Jessika Bertacchini, Diego Angeli

**Affiliations:** Dipartimento di Ingegneria Enzo Ferrari, Università degli Studi di Modena e Reggio Emilia, Modena 41125, Italy; Centro Interdipartimentale per la Ricerca Applicata e i Servizi nella Meccanica Avanzata e nella Motoristica InterMech-MO.RE, Piazzale Europa, 1, Reggio Emilia RE 42124, Italy; Department of Sciences and Methods for Engineering, University of Modena and Reggio Emilia, Reggio Emilia 42122, Italy; Dipartimento di Ingegneria Enzo Ferrari, Università degli Studi di Modena e Reggio Emilia, Modena 41125, Italy; Dipartimento di Ingegneria Enzo Ferrari, Università degli Studi di Modena e Reggio Emilia, Modena 41125, Italy; Dipartimento di Ingegneria Enzo Ferrari, Università degli Studi di Modena e Reggio Emilia, Modena 41125, Italy; Institute of Science and Technology for Ceramics (ISTEC), National Research Council (CNR), Faenza 48018, Italy; Department of Surgery, Medicine, Dentistry and Morphological Sciences with Interest in Transplant, Oncology and Regenerative Medicine, University of Modena and Reggio Emilia, Modena 41125, Italy; Department of Surgery, Medicine, Dentistry and Morphological Sciences with Interest in Transplant, Oncology and Regenerative Medicine, University of Modena and Reggio Emilia, Modena 41125, Italy; Istituto di Genetica Molecolare “Luigi Luca Cavalli-Sforza”, Consiglio Nazionale della Ricerca (IGM-CNR), Bologna 40136, Italy; Department of Sciences and Methods for Engineering, University of Modena and Reggio Emilia, Reggio Emilia 42122, Italy

**Keywords:** experimental, CFD, modellings, hydroxyapatites, scaffolds, biomaterial cell interaction, bone, regenerative mechanism

## Abstract

In the field of bone tissue engineering, particular interest is devoted to the development of 3D cultures to study bone cell proliferation under conditions similar to *in vivo* ones, e.g. by artificially producing mechanical stresses promoting a biological response (mechanotransduction). Of particular relevance in this context are the effects generated by the flow shear stress, which governs the nutrients delivery rate to the growing cells and which can be controlled in perfusion reactors. However, the introduction of 3D scaffolds complicates the direct measurement of the generated shear stress on the adhered cells inside the matrix, thus jeopardizing the potential of using multi-dimensional matrices. In this study, an anisotropic hydroxyapatite-based set of scaffolds is considered as a 3D biomimetic support for bone cells deposition and growth. Measurements of sample-specific flow resistance are carried out using a perfusion system, accompanied by a visual characterization of the material structure. From the obtained results, a subset of three samples is reproduced using 3D-Computational Fluid Dynamics (CFD) techniques and the models are validated by virtually replicating the flow resistance measurement. Once a good agreement is found, the analysis of flow-induced shear stress on the inner B-HA structure is carried out based on simulation results. Finally, a statistical analysis leads to a simplified expression to correlate the flow resistance with the entity and extensions of wall shear stress inside the scaffold. The study applies CFD to overcome the limitations of experiments, allowing for an advancement in multi-dimensional cell cultures by elucidating the flow conditions in 3D reactors.

## Graphical Abstract

**Figure:**
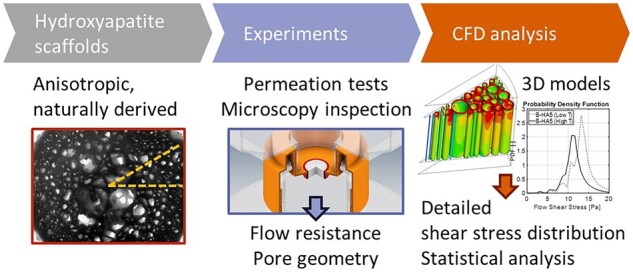

## Introduction

Bone regeneration is a well-orchestrated physiological process, which takes place during normal fracture healing, and which is involved in continuous remodelling throughout adult life. Bone can be seen as a material composed of collagen fibres, crystals of hydroxyapatite (HA), an extracellular matrix (ECM), growth factors and a complex vascular system. The cells that make up the bone include the osteoprogenitor cells, i.e. osteoblasts and osteocytes, and bone-resorptive cells, i.e. osteoclasts. Bone regeneration requires three key components: an osteoinductive signal, an insoluble substratum that delivers the signal and acts as a scaffold for the induction of new bone formation, and host cells that are capable of differentiation into bone cells in response to the osteoinductive signals. Bone tissue engineering (BTE) is aimed to complete repair and regenerate bone defects that fail to spontaneously heal, through new and versatile strategies [1].

In the field of *in vitro* tissue culturing, 3D scaffolds are receiving remarkable attention [2, 3] to promote the cells growth and differentiation in an *in vivo*-like environment. In this context, both hydrogel and HA are the most established materials for living cell support, sharing the aim to mimic the ECM of the bone tissue, i.e. a nontoxic and functional vascularized medium for *in vitro* studies on bone regeneration. Hydrogels are synthetic polymeric materials with excellent biocompatibility, creating a hydrophilic 3D environment suitable to cell survival and proliferation [4, 5], whereas HA scaffolds mimic natural bone characteristics, matching chemical–physical composition and 3D porous structure, that allow for cell mobility metabolic processes [1].

These must be embedded in an externally controllable system for nutrients delivery, waste removal and the generation of mechanical stimuli. This last aspect proved important to promote growth processes (e.g. osteogenesis) via the induction of biological stimuli in response to mechanical solicitation (mechanotransduction) [6]. In bone remodelling applications, it is therefore of primary importance to replicate the matrix and interstitial flows in the bone porous lacunae induced by muscle contraction and gravitational forces [7]. Therefore, BTE using dedicated bioreactors able to reproduce a predefined range of mechanical forces (e.g. flow-induced shear stress, FSS) is of utmost interest, since it overcomes the intrinsic limitations of static cultures. Several perfusion-based bioreactors for cell proliferation were presented by Vukasinovic *et al*. [8], Kim and Ma [9] and Zhao *et al*. [10].

From the standpoint of fluid dynamics, this kind of flow falls in the branch of microfluidics, i.e. the study through simulations of fluids at submillimetre scales [11]. Several examples of microfluidics-based studies dealt with the flow-induced biological response of cells [12, 13], especially focusing on 3D tissue processes although the poor degree of flow control and the experimental-only investigation hindered a full comprehension of the flow-biological interaction [14].

In the authors’ opinion, the wide potential of microfluidic application for tissue engineering has been underexplored by the still limited use of multi-dimensional Computational Fluid Dynamics (CFD) techniques, whose investigation potential at ultra-small scales and the reduced cost make it a very powerful tool. Examples of the pioneering application of CFD to biological systems are the computational study of the flow-functional skeleton of the deep-sea glass sponge *Euplectella aspergillum* [15], the quantification of the centrifugal force effect on insect cell line [16], the shear stress generated by air bubble aerator [17] and the FSS quantification of Ti/HA on two porous scaffolds [18]; in this broad spectrum of cases, CFD allowed for the detailed study of aspects difficult (or even impossible) to be directly observed in experiments. Focusing on tissue regeneration, earliest studies characterized the flow in isotropic scaffolds using CFD, such as the analyses of periodic homogeneous structures from Boschetti *et al*. [19] and Lesman *et al*. [20]. The major limitation of such studies was the reproduction of a porous isotropic structure for cell proliferation, whereas the marked direction-dependent properties of many living tissues alter the media diffusion and the nutrient delivery.

In this article, a simulation study is presented on 3D models of anisotropic HA scaffolds exposed to gravity-induced flow. Preliminary experiments are performed on real specimens of a specific type of HA, the B-type (B-HA), obtained by synthesis of natural species [21]. Geometrical data on the pores and the flow resistance of the scaffolds are measured by means of microscopy inspection and permeation tests as necessary parameters for the calculation and control of FSS levels in perfusion bioreactors. The difficulty to characterize the non-repeatable features of B-HA samples is highlighted by the largely different experimental outcomes. Such an engineering challenge is tackled by virtually designing a dataset of three specimens based on real counterparts that show clearly distinct flow resistances, and using these models for a computational study using 3D-CFD. After an initial validation against the corresponding measured data, the models are used to investigate FSS values on the inner scaffold structure. This includes: (i) a spatial statistical analysis on FSS ranges, (ii) the derivation of a global simulation-based expression and (iii) a contribution to the understanding and advancement of the biologic-scaffold interaction for bone tissue regeneration.

## Experimental methods

A detailed characterization of the biomaterial in use is needed for the knowledge of the fluid–biological interaction described herein. Three-dimensional HA scaffolds are investigated in this work; the material is obtained via a biomorphic transformation of natural wood that results in B-type carbonation (B-HA) [21]. Excellent mechanical and osteoconductive properties were observed for this material, which appears a promising candidate for bone regenerative purposes [22–24]. A set of 14 cylindrical B-HA specimens is available for this project (named B-HA*x*, with *x* being the specimen number), with diameters in the range 10.4–10.9 mm and axial length between 3.7 and 4.8 mm. B-HA presents a hollow micro-tubular structure, faithfully representing the mineralized bone tissue; the pores can be considered as roughly parallel but (i) with inherent poor repeatability due to the production process and (ii) with possible internal interconnections between adjacent channels. Both aspects prevent the introduction of simplified expressions to calculate the fluid dynamic properties of B-HA, which look specific to the individual specimens. Consequently, a direct measurement of the scaffold flow resistance is required, together with the pore geometry, in order to estimate the FSS acting on cells during biological tests in perfusion bioreactors.

**Figure 1.**
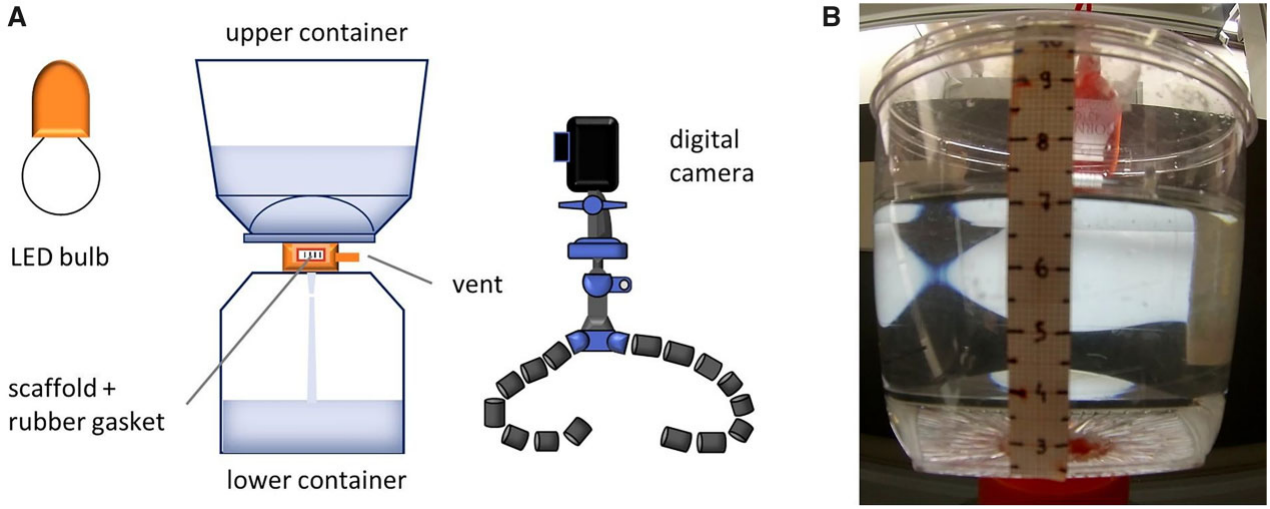
(**A**) Sketch of the experimental apparatus used in permeation tests. (**B**) Example of an acquired frame (room temperature).

The first goal is accomplished here by performing permeation tests with a simple experimental apparatus, consisting, basically, of two transparent containers at ambient pressure and an imaging system (GoPro Hero3 and a LED back lighting), as depicted in Fig. 1A. An orifice connecting the two stacked tanks hosts the B-HA sample, whose sealing is ensured by a silicone rubber gasket. A given volume of liquid (500 ml of bi-distilled water) is poured in the upper container and drains through the scaffold under gravity driven flow. Optical acquisitions in time-lapse mode allow for the measurement of the fluid level variation; in Fig. 1B, one of the frames of a saved image sequence is reported as an example. In this way, the pressure drop over the scaffold (Δp) and the volumetric flow rate ($Q_{v}$) can be expressed in terms of the instantaneous liquid height (h(t)) (Equations (1 and 2), respectively):

(1) Δp=ρgh(t),

(2) $$Q_{v}=\frac{\partial V}{\partial t}=-\pi \frac{D^{2}}{4}\frac{\partial h}{\partial t},$$

where ρ is the fluid density and D is the instantaneous diameter of the liquid free surface, which is a function of the liquid height, too. The flow resistance ($R_{f}$) at a given instant is then calculated, after rearranging and integrating Equation (3) between the initial frame and the time considered; an average value is finally obtained from the data relative to the single frames. More details about the experimental set-up and data extraction are reported in Salerno *et al*. [25].

(3) $$R_{f}=\frac{\Delta p}{Q_{v}}.$$

Possible variations in the scaffold response with temperature are considered by replicating the experiments in two ambient conditions: (i) at room temperature and (ii) at 37°C, i.e. the temperature commonly applied for cell culturing in incubators. In the latter case, the entire apparatus is transferred inside an oven. The fluid temperature is registered from thermocouple readings before and after each acquisition. For each specimen, 4–5 consecutive repetitions are performed at both conditions.

**Figure 2.**
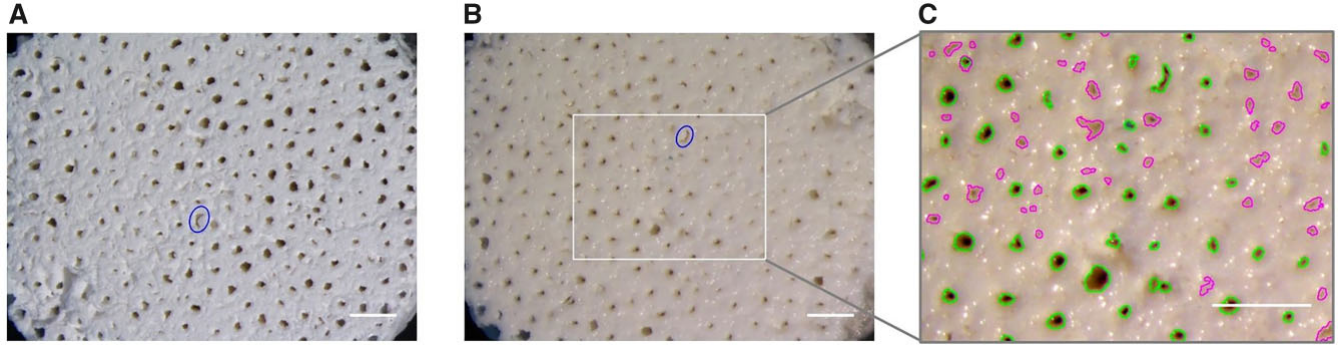
Microscope images representing the same face of a sample (B-HA13) (**A**) before and (**B**) after exposure to water flow, with reduced pore size in (B) due to water absorption by the sample material. In image (B), the sample is rotated of nearly 180° in the figure plane (one of the pores is indicated for reference). The enlargement in (**C**) shows the pores identified by the double processing procedure. Scale bar: 1 mm.

Data relative to the number and size of the pores are retrieved via a visual examination of all the B-HA samples, including microscope acquisitions of both front and rear faces (stereoscope Nikon SMZ800, New York, USA). Preliminary observations indicate that B-HA is prone to swelling as a consequence of fluid immersion, as demonstrated in Fig. 2A and B, which portray the same specimen before and after the experiments. For this reason, the morphological analysis of each sample is carried out immediately after the relative cycle of permeation tests is completed, in order to relate the extracted flow resistances to the actually encountered pore geometry. Microscope images are processed with the software ImageJ (NIH, USA); the processing procedure consists of three steps: (i) filtering, (ii) thresholding, aimed at separating the features of interest (i.e. the pores) from the background and (iii) the subsequent application of a native automated technique for particle detection, which identifies the individual pores and outputs their cross-sectional area. Data extraction is repeated two times on both sides of the scaffolds by adjusting the threshold value and the parameters for particle detection. Both large and small pores, of section as small as 47 μm^2^, can be thus properly handled while limiting area over-/under-estimation connected to improper thresholding. Attention is paid in avoiding duplicate counts. An example of the detections generated by this procedure is illustrated in Fig. 2C. Equivalent diameters are derived from the measured cross-sections, and are used to cluster pores in discrete classes.

## Experimental results

**Figure 3.**
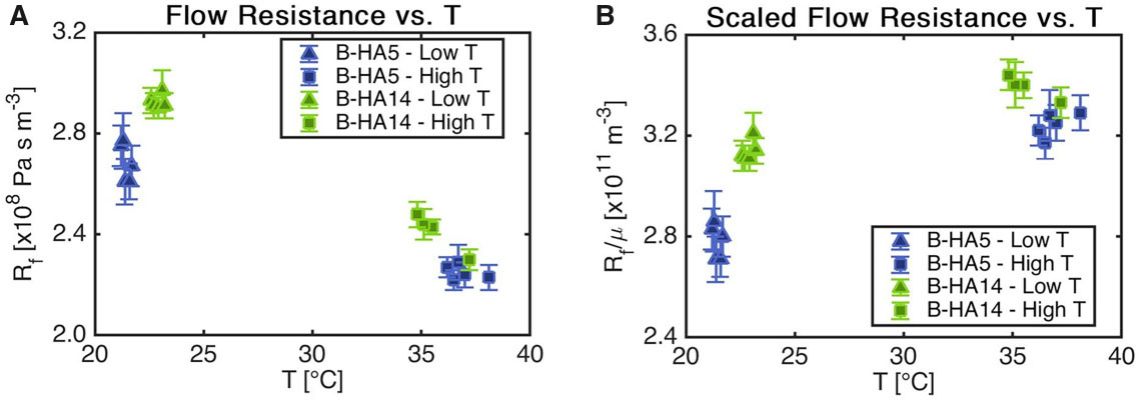
Plots of (**A**) flow resistance and (**B**) flow resistance-to-viscosity ratio as a function of the fluid temperature for two samples (B-HA5 and B-HA14). The test sequence for B-HA14 (High T, Low T) is opposite with respect to B-HA5 (Low T, High T).

Results relative to B-HA flow resistances are exemplified in Fig. 3. Average values of $R_{f}$ from single permeation tests are plotted in Fig. 3A for two samples, namely B-HA5 and B-HA14, as a function of the mean liquid temperature. Datasets corresponding to the two different ambient conditions are distinguished. It is pointed out that experiments at room temperature are performed first in the case of B-HA5; the test sequence is reversed for B-HA14. In both cases, tests at 37°C denote a larger scattering for the liquid temperature with respect to tests at room temperature, since water transfer operations from the lower to the upper container require the assembly to be extracted from the oven at the end of each acquisition (the same volume of liquid is used in consecutive tests). Nevertheless, for each specimen, resistance values are quite consistent within each set, whereas they show a net decrease when moving to higher temperatures.

In order to clarify the observed trend and with the prospect of exposing the scaffolds to fluids other than water, i.e. culture medium, the flow resistance is scaled by the liquid properties. In particular, for laminar flow conditions, as in the present problem, the Darcy’s law could be retained valid, so the following expression applies [26]:

(4) $$R_{f}=\;\frac{L\;\mu }{A\;k}.$$

In Equation (4), L, A and k are intrinsic of the material, describing the height, cross-sectional area and permeability of the scaffold, respectively, while μ is the dynamic viscosity of the liquid, which holds the dependency on temperature. Hence, the $R_{f}$-to-μ ratio is calculated, by considering temperature-varying water viscosity.

In Fig. 3B, it is observed that $\frac{R_{f}}{\mu }$ still preserves a dependency on the liquid temperature, contrary to what expected. Larger values are found for all the specimens, corresponding to the set of tests in the oven. Such a behaviour cannot be ascribed to an increased swelling of the scaffolds consequent to a longer fluid exposure, as this was excluded by reversing the order of the tests. Instead, it is more likely that the conditions for the validity of the Darcy’s law are not fully satisfied, and temperature indirectly affects flow development inside the pores. This consideration will be better discussed in the following paragraphs in light of further results. For now, a weighted average is performed on the data extracted from single tests to provide a unique value of $\frac{R_{f}}{\mu }$ for each specimen, useful for the computation of FSS in bioreactor experiments as proposed in Salerno *et al*. [25]. The results are reported in Table 1 for all the samples. A wide variety of values is obtained, ranging approximately from 2 to 30 × 10^11^ m^−3^.

**Table 1.** Average flow resistances (scaled) and information on pore geometry for the investigated scaffolds

	***R_f_*/*μ***[×10^11^ m^–3^]	**Pore nr.** [–]	***D*_max_** [μm]	***D*_mean_** [μm]		***R_f_*/*μ***[×10^11^ m^–3^]	**Pore nr.** [–]	***D*_max_** [μm]	***D*_mean_** [μm]
**B-HA1**	2.67 ± 0.12	307/410	325/337	146	**B-HA8**	13.9 ± 1.4	568/526	298/303	107
**B-HA2**	6.2 ± 0.6	710/607	405/626	132	**B-HA9**	6.7 ± 0.5	434/373	706/521	125
**B-HA3**	12.06 ± 0.06	938/862	383/500	97	**B-HA10**	2.4 ± 0. 2	489/393	666/820	130
**B-HA4**	4.1 ± 0.2	545/589	405/581	125	**B-HA11**	6.9 ± 0.3	450/435	321/378	106
**B-HA5**	3.0 ± 0.2	527/561	910/860	151	**B-HA12**	1.7 ± 0.2	423/439	618/632	156
**B-HA6**	27 ± 3	598/909	178/178	60	**B-HA13**	9.6 ± 0.3	372/515	283/311	107
**B-HA7**	4.2 ± 0.3	567/537	386/437	115	**B-HA14**	3.26 ± 0.13	494/412	283/264	108

The number and maximum diameter of the pores are distinguished for the two sides of the samples.

**Figure 4.**
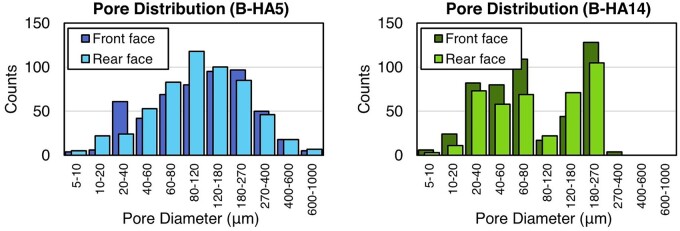
Histograms of pore distribution for B-HA5 and B-HA14.

Such a large spread is directly connected to the natural origin of B-HA, which implies a variable and non-controllable pore distribution. The analysis on the front and rear sides of the scaffolds reveals the presence of hundreds of pores, with diameters covering three orders of magnitude: pores as small as 8 μm in diameter are found in all the specimens, while the maximum size varies in-between 180 and 910 μm. Statistical data describing scaffold morphology are appended in Table 1. No correlations can be delineated between these quantities and the measured flow resistances: an evidence is provided by the very two samples discussed above, which have a similar $\frac{R_{f}}{\mu }$ in spite of different pore geometries. In order to interpret the fluid dynamic behaviour of the samples, pore distribution must be considered in more detail, as offered by the histograms in Fig. 4. Large pores (400–1000 μm) in specimen B-HA5 are responsible for its relatively low resistance; in B-HA14, these classes of size are absent but compensated by the position of the peak, which is shifted towards greater diameters with respect to B-HA5. In all the investigated specimens, the front and rear faces present a similar pore number and distribution, confirming the almost unidirectional hierarchical organization of B-HA. This allows for the modelling of each sample as a network of parallel cylindrical channels, with various diameters.

## Numerical model

The measured data for scaffold flow resistance and pore geometry allow for the computation of fluid shear stresses acting on the deposited cells during *in vitro* experiments in bioreactors [25]. However, these are in the form of average quantities only. To investigate in detail the FSS conditions experienced by the cells, the fluid dynamic behaviour of B-HA is further explored in this paper by means of CFD simulations.

Based on the experimental observations, a selection of three B-HA samples is considered for the numerical analyses. A preliminary validation of the scaffold–fluid interaction is performed, followed by an in-depth investigation of FSS. A 30° angular sector of each sample is considered in order to reproduce the sample-specific diameter distribution observed in the experiments, while preserving a high computational efficiency. This allows for the reproduction of an analogous pore distribution as the full-scale counterpart, with a relevant reduction of the overall simulated domain (in this case 1/12). This modelling choice has been considered adequate to preserve the channel size distribution measured in the original samples, with a simultaneous limitation of the associated computational cost. Each simulated B-HA sample preserves the specimen-specific diameter and axial length. The simulated domain extension follows the guidelines proposed in Maes *et al*. [18], although here a highly preferential (axial) canalicular structure is evident.

**Figure 5.**
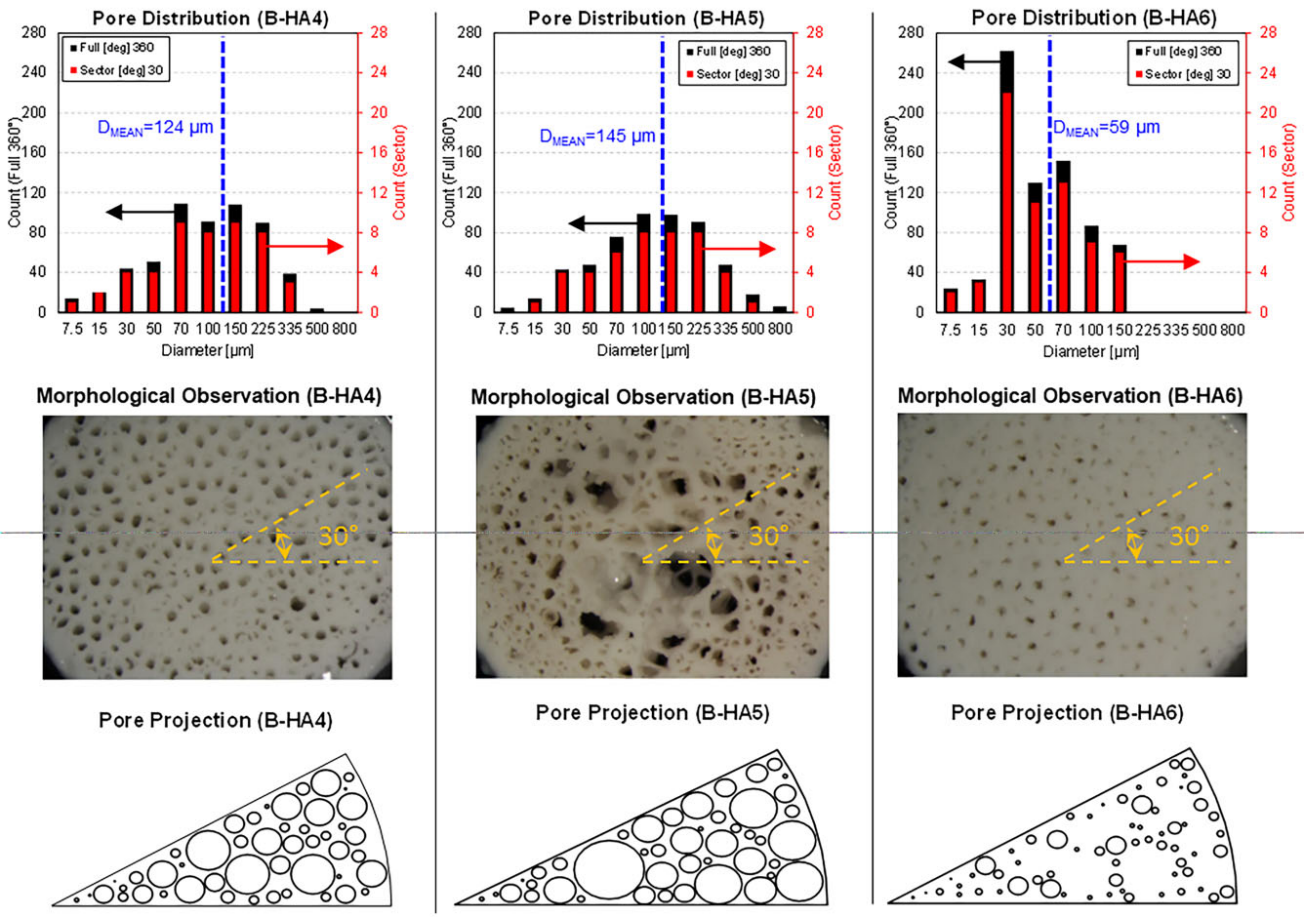
Pore distribution for the full-scale and 30° sector (top), comparison with the visual acquisition (middle row) and 2D sketch for 3D-CFD model creation (bottom) for B-HA4 (left), B-HA5 (Middle column) and B-HA6 (right).

The clustering of the pore diameters for the three samples is reported in Fig. 5 (top) for the full-size and the 30° sector, including the pore count for both. In Fig. 5 (bottom), the resulting 2D sketch is displayed, which will be used for the creation of the 3D model.

The model creation and simulation are carried out using SIMCENTER STAR-CCM+ v2021.3 licensed by SIEMENS SISW. Three independent models reproducing the selected samples (B-HA4/5/6) are created, with case-specific differences consisting of (i) the pore distribution reported in Fig. 5, (ii) the sample geometrical dimensions and (iii) the applied pressure differential, as listed in Table 2. For simplicity, the imposed pressures correspond to experiments performed at constant hydraulic head, as specified in Salerno *et al*. [25]. Porosity values (i.e. the ratio of void over the total volume) for the three B-HA samples are also added.

**Table 2.** Main dimensions, pressure and temperature measured in the experiments and used in 3D-CFD simulations

	Diameter × axial length [mm]	Porosity [–]	Pressure differential [Pa]	Temperature [°C]
**B-HA4**	10.4 × 4.3	0.48	947	*T* = 22
				*T* = 37
**B-HA5**	10.6 × 3.9	0.60	947	*T* = 21
				*T* = 37
**B-HA6**	10.7 × 3.8	0.13	458	*T* = 22
				*T* = 35

All models include a multi-physics approach, with the solid physics pertaining to the scaffold structure and the liquid one reproducing pure water, with temperature-dependent material properties. Given the generally low flow velocity, a laminar flow model is adopted. Two test temperatures are considered for each case, ∼21°C/22°C (named *Low T*) and 35°C/37°C (named *High T*), the exact temperatures slightly varying to replicate the experimental conditions. For the same reason, the pressure differential applied to reproduce the constant hydraulic head acting on each tested sample varies, with the outlet section being at ambient pressure for all cases.

**Figure 6.**
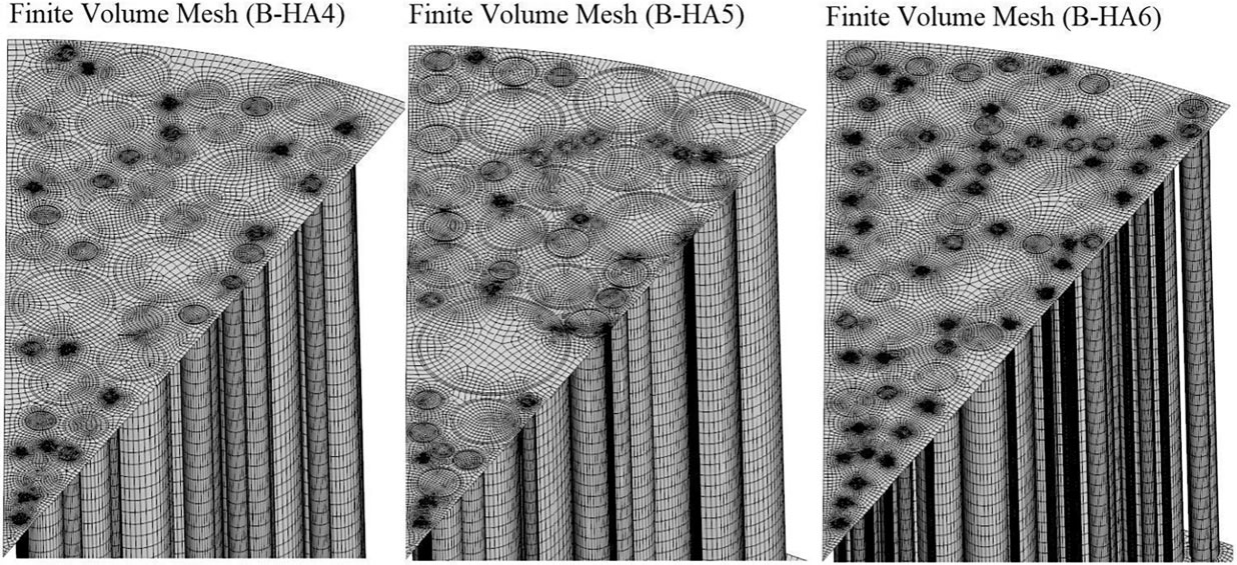
Finite volume mesh at the interface between scaffold/plenum and along the channel walls for B-HA4 (left), B-HA5 (Middle) and B-HA6 (right) models.

A particular care is devoted to the generation of the finite volume mesh, in view of the peculiar geometrical characteristics of the analysed samples. A structured channel-oriented (i.e. axially directed) hexahedral mesh is used for all cases, sharing the same mesh setup (hexahedral mesher with 0.06 mm surface size, uniformly-spaced 1 mm cell size in the channel direction and a prism layer thickness differentiated according to channel diameter, from 1 μm to 10 μm) and adding two fluid plenums at the top/bottom of the B-HA samples (1 mm extended in the axial direction) for numerical stability. Axially directed extruded grids are created also for the plenums, with 0.5 mm uniformly-spaced hexahedral cells. The inclusion of plenums in the simulated fluid domain allows for the reproduction of the natural preferential flow repartition towards larger channels (i.e. reduced flow resistance). The two types of meshes (i.e. the channel region and top/bottom plenums) are matched by means of internal interfaces, allowing a complete flow/thermal coupling between the different fluid regions. The pore distribution of each model follows that reported in Fig. 5 (bottom), which is used to generate the sample-specific surface mesh from which volumetric extrusion led to the creation of the channels volume grid. In Fig. 6, a close-up of the three generated finite volume meshes is reported, highlighting the channel-directed extrusion used for the mesh creation for the channels from the experimentally derived pore distribution. The total number of finite volume cells varies between 1.5 and 3 M for the full model (fluid and solid parts). Stagnation inlet and pressure outlet boundary conditions are applied at the upper/lower plenums, and no-slip conditions are imposed at the fluid–solid interface of the scaffold channels and faces. The use of no-slip boundary conditions for solid walls is motivated by the lack of direct measurements of the actual wall roughness in the scaffold channels. This constitutes an aspect of necessary improvement for future developments, both in terms of biological effect on cell adhesion, and in terms of model implementation. A steady-state solution solver is used, with discretization schemes using second-order accuracy.

**Figure 7.**
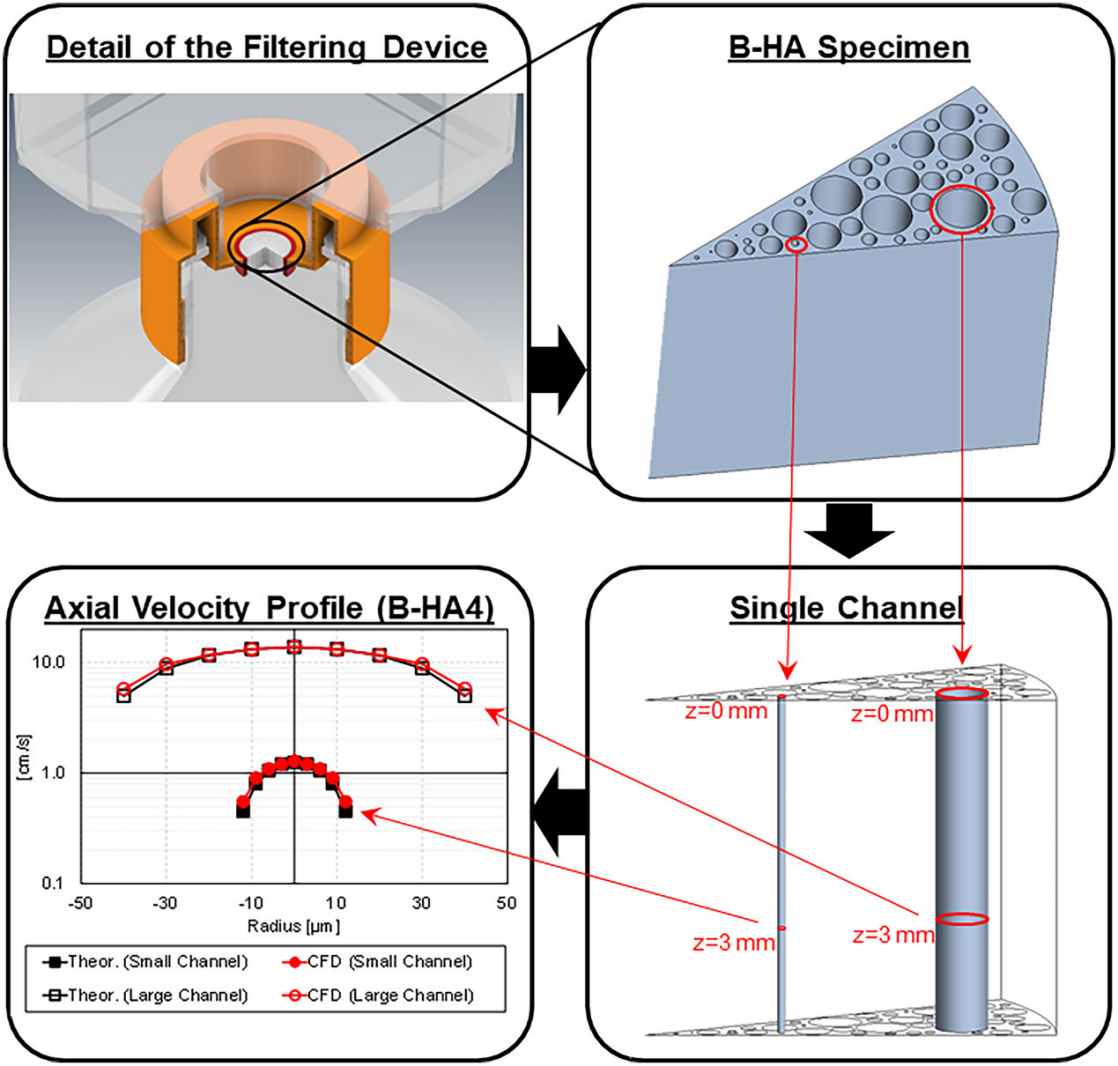
Analysis operations and axial velocity profiles (logarithmic scale) for a small (*D* = 30 μm) and a large (*D* = 100 μm) channel.

Mesh quality is assessed by extracting axial velocity profiles on two representative channels. The laminar flow in a cylindrical channel is a canonical case (Hagen–Poiseuille) for which the analytical solution in Equation (5) indicates a parabolic-type axial velocity $u_{z}(r)$ profile in cylindrical coordinates for the i-th channel (with $r_{i}$ radius). In Fig. 7, the analysis operations are reported, with axial velocity profiles on an 11-point transverse probe line for a small (*D* = 30 μm) and a large channel (*D* = 100 μm) at 3 mm from the inlet section. Both simulation results are in good agreement with the analytical profile, thus confirming the adequacy of the mesh quality in both small and large channels.

(5) $$u_{z}\left(r\right)=-\frac{\partial p}{\partial z}\frac{\left(r_{i}^{2}-r^{2}\right)}{4\mu }.$$

## Numerical results

### Flow resistance

**Figure 8.**
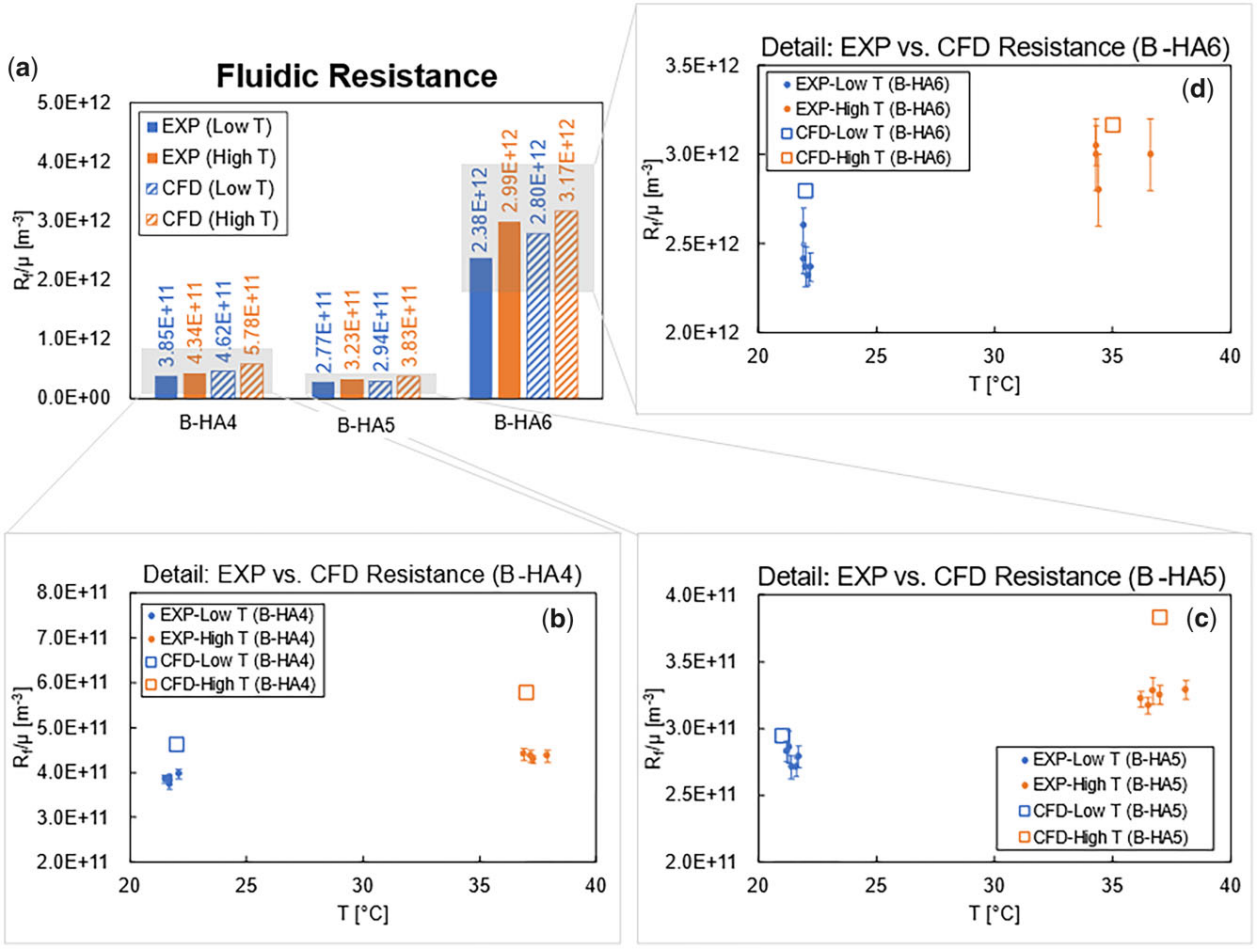
Comparison of experimental and simulated values of normalized flow resistance $\frac{R_{f}}{\mu }$: (**a**) average values, and test-individual measurements for B-HA4 (**b**), B-HA5 (**c**) and B-HA6 (**d**).

The first observed result from the simulations is the normalized flow resistance $\frac{R_{f}}{\mu }$, where *μ* is the fluid dynamic viscosity and $R_{f}$ is calculated from the simulation results as in Equation (3), and whose values are compared with the experimentally measured data for model validation. In Fig. 8A, the values for the three samples at the two tested temperatures (Low/High T) are reported and compared to the average of the experimental runs. For all cases, it is noted that (i) an increase in the normalized flow resistance is always observed from low to high temperature, and (ii) the entity of the flow resistance variation among the three selected samples largely differs, with low/very low/high flow resistance for B-HA4/5/6, respectively. Both aspects are well reproduced by the CFD models, with a resistance overestimation visible in all the cases, possibly due to the non-permeability of solid scaffold in the models, whose modelling will be investigated in future steps. The close-ups showing the experimental variability between consecutive tests (Fig. 8B–D) confirm the absence of evident outlier acquisitions and the accuracy of the values predicted by the CFD simulations.

**Figure 9.**
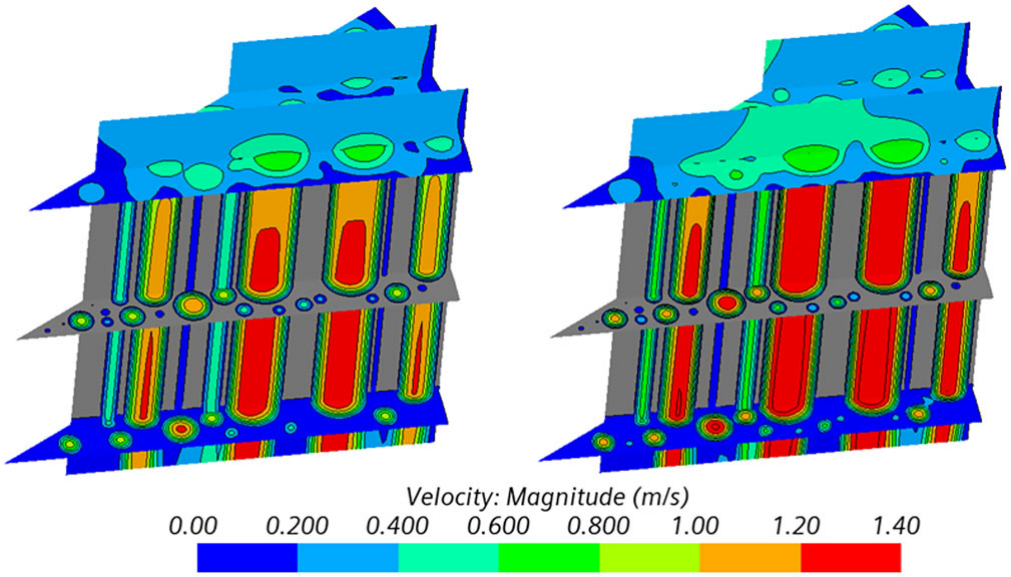
Velocity magnitude at low temperature (left column, *T* = 22°C) and high temperature (right column, *T* = 37°C) for B-HA4. Vertical scale bar: 1 mm.

The generally higher value for the normalized flow resistance $\frac{R_{f}}{\mu }$ is explained by the water dynamic viscosity reduction with temperature, varying from ∼1 × 10^−3^ Pa·s to 0.719 × 10^−3^ Pa·s for 20°C and 35°C, respectively. This has a direct effect on the flow velocity as well as on the entry region for the complete development of the velocity profile, as illustrated in Fig. 9 where the velocity magnitude in B-HA4 for both the simulated fluid temperatures is compared. The contours highlight that the length of the entry region is not negligible for these samples, being of nearly 1 mm against 4–5 mm of scaffold total height; as a consequence, the assumption of fully developed flow, which the Darcy’s law relies on, cannot be considered completely fulfilled for the present problem. In addition, an increase in the entry region length with temperature is observed, as a result of the lower fluid viscosity and higher velocity values, thus corroborating the considerations already brought forward in light of the experimental results.

### Flow shear stress

**Figure 10.**
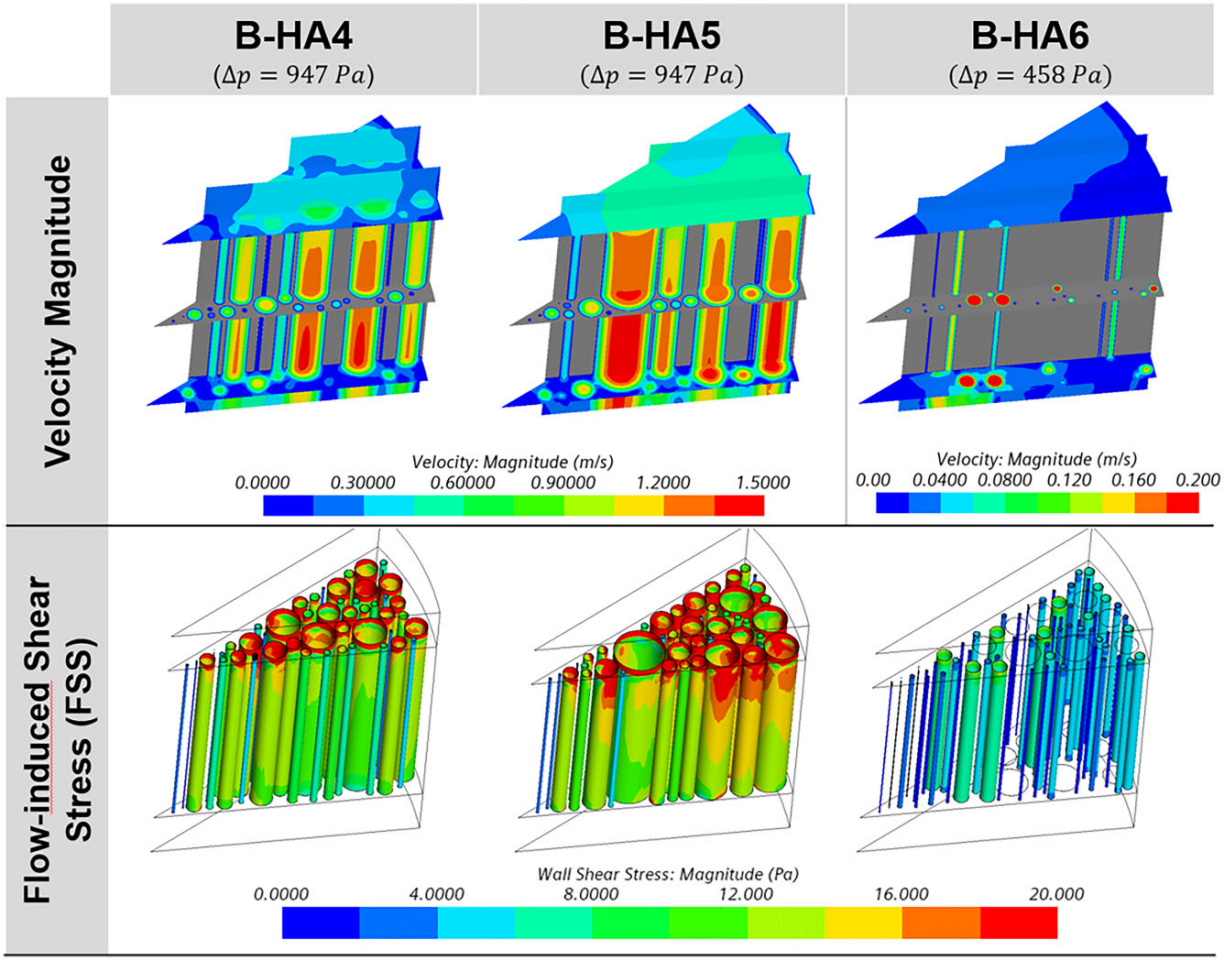
Velocity magnitude on three sections (top) and FSS fields at scaffold–fluid interfaces (bottom) for B-HA4 (left), B-HA5 (middle) and B-HA6 (right) at 21/22°C (from Table 2).

**Figure 11.**
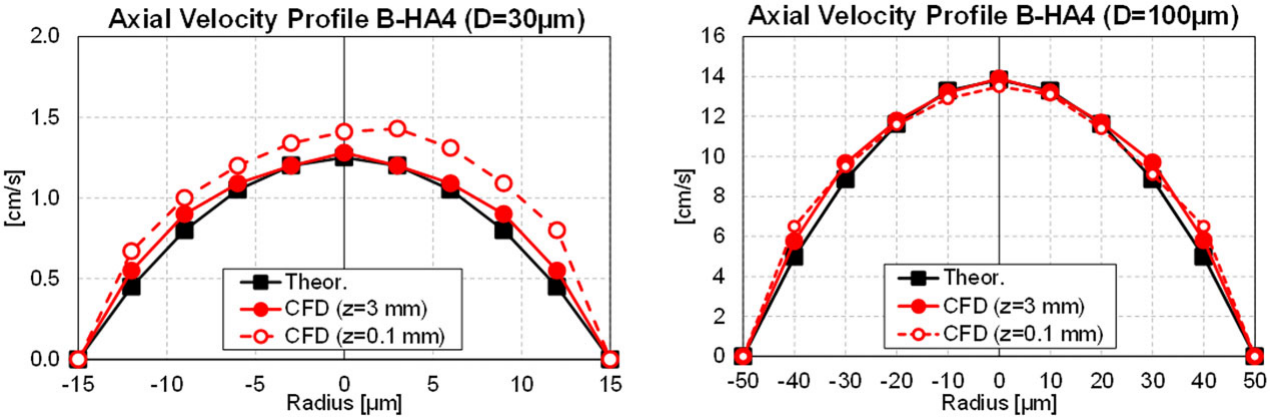
Axial velocity profiles for a small (*D* = 30 μm) and a large (*D* = 100 μm) channel inside the scaffold (*z* = 3 mm) and close to the channel inlet (*z* = 0.1 mm).

The second observed result is the average value and the distribution of the simulated FSS. Being the wall stresses the sole responsible for the validated flow resistance, it is inferred that also the entity of the FSS accurately reproduces the physical counterpart. This overcomes the high difficulty associated with a direct measurement of such a quantity. In Fig. 10, the velocity magnitude and the FSS at pore walls are reported, showing (i) a qualitative proportionality with the pore diameter (i.e. higher FSS in large pores) and (ii) a peak FSS region in the proximity of the upper inlet region, due to the development of the velocity boundary layer. This second aspect can be explained by comparing the axial velocity profiles ($u_{z}$) for the two channels considered in Fig. 7 (located at 0.1 and 3 mm from the inlet section), which reveal a higher velocity gradient at the wall in the upper position (Fig. 11). This justifies the higher FSS value observed near the channel inlet, also implying that cells deposited in this region are exposed to larger FSS than theoretically expected. It is also worth observing that the velocity profiles at 0.1 mm are not parabolic, which is explained by the disturbance effect of the flows in adjacent channels. These effects are strongly channel-dependent, given the random location and size of adjacent channels, and they are reduced far from the channel inlet, as the parabolic velocity profiles at 3 mm confirm.

**Figure 12.**
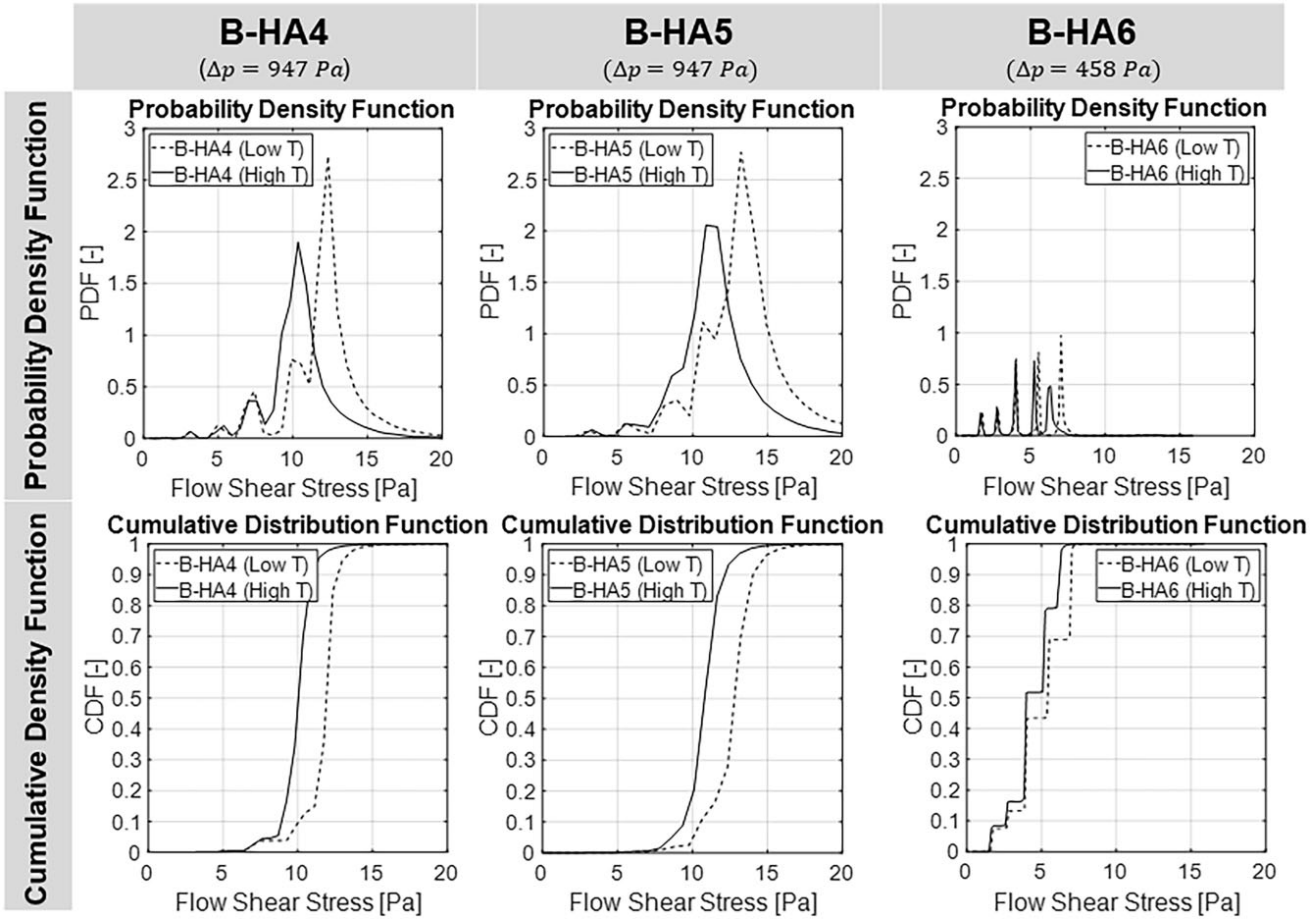
Probability Density Functions (PDFs) and normalized Cumulative Density Functions (CDFs) for B-HA4 (left), B-HA5 (middle) and B-HA6 (right).

More quantitative analyses regarding shear stress at the scaffold inner walls are carried out by extracting FSS values and constructing discrete Probability Density Functions (PDFs) for the FSS (Fig. 12, top). For this purpose, the FSS range is discretized in 100 bins, counting each bin contribution (${P|}_{i\leq {\mathrm{FSS}}_{k}<i+1}$) in the range $i\leq {\mathrm{FSS}}_{k}<i+1$ as in Equation (6), with $A_{k}$ being the surface area subject to the ${\mathrm{FSS}}_{k}$ shear stress. The normalized integral (i.e. the Cumulative Density Function, CDF) provides the overall FSS repartition within the sample, as reported in Fig. 12 (bottom).

(6) $${P|}_{i\leq {\mathrm{FSS}}_{k}<i+1}=\frac{\sum_{k=i}^{i+1}{\mathrm{FSS}}_{k}A_{k}}{\sum_{k=i}^{i+1}A_{k}}.$$

It is to be underlined that such FSS values are relatively high compared to physiological FSS ranges (in the order of 0.1–1 Pa) for cell culture growth: this is motivated by the use of water as a fluid (both in experiments and in simulations), instead of solute-rich culture media (e.g. DMEM). As measured by Poon [27], both density and molecular viscosity of culture media are significantly higher than distilled water (up to 60%), and the difference increases for solutions with nutrient content as high as 20% by volume.

**Figure 13.**
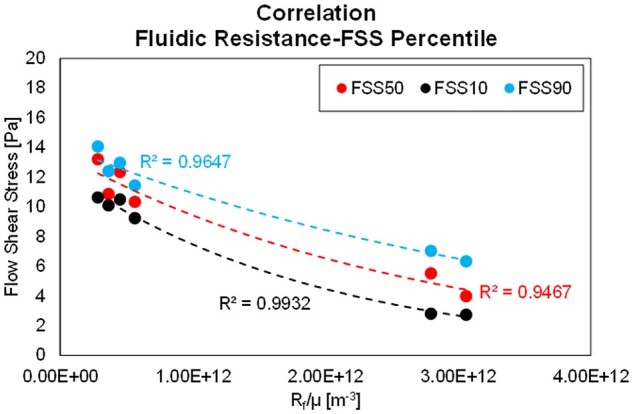
Simulation results and exponential fits for FSS10, FSS50 and FSS90 as a function of the normalized flow resistance $\frac{R_{f}}{\mu }$, as reported in Equations (3–5).

Based on the CDFs reported in Fig. 12, statistical considerations are possible on the degree of FSS experienced by the cells deposited on each sample, e.g. the FSS value acting on the 10%, 50% or 90% of the overall scaffold inner surface. This might be largely different with respect to the average value, and it directly stems from both the impossibility to realise a good repetition of pore sizes in the B-HA bio-derived material and the entry region for the flow profile development. The presented engineering method is explicitly developed to accept the biomaterial irregularity and its finite axial extension, allowing to relate the fluid dynamic behaviour of the sample (e.g. the global flow resistance) with a statistical-based range of flow-induced stress. In Fig. 13, a correlation analysis is carried out between the FSS acting on the 10%, 50% and 90% of the scaffold surface (FSS10, FSS50 and FSS90, respectively) for the three tested samples, each at two temperature levels. Despite the relatively low number of tested samples, their largely different flow resistance is used to obtain exponential-type trend lines for each FSS percentile, describing the non-linear relationship between flow resistance and FSS percentiles.

Based on these outcomes, a generic B-HA relationship between flow resistance and the entity of FSS experienced by the deposited cells is obtained, which can be generalized for this type of B-HA scaffold. The derived exponential expressions for FSS10, FSS50 and FSS90 are reported in Equations (7–9), respectively. These can be used to correlate a simple flow resistance test for a generic B-HA sample (hence measuring $\frac{R_{f}}{\mu }$) with the presumed area percentage subject to FSS levels of interest with a good approximation.

(7) $$\mathrm{FSS}10\left(\frac{R_{f}}{\mu }\right)=12.5\cdot e^{-5.03E-13\cdot \frac{R_{f}}{\mu }\;}.$$

(8) $$\mathrm{FSS}50\left(\frac{R_{f}}{\mu }\right)=13.654\cdot e^{-4E-13\cdot \frac{R_{f}}{\mu }\;}.$$

(9) $$\mathrm{FSS}90\left(\frac{R_{f}}{\mu }\right)=14.167\cdot e^{-3E-13\cdot \frac{R_{f}}{\mu }\;}.$$

The cross-disciplinary biological/engineering topic, and the experimental/simulation method presented, potentially unlock innovative engineering possibilities in the design of bio-derived materials for bone regeneration. These outcomes relevantly extend the understanding the scaffold–fluid interaction, thus providing useful considerations usable at a design stage. A further development of the study will deal with an optical-based characterization of the FSS percentile correlation, in order to be able to provide similar information solely based on sample photographs.

## Conclusions

In this study, an experimental and numerical research is carried out on the use of multi-dimensional scaffolds for bone cells proliferation. HA-based scaffolds (B-HA) is chosen to reproduce the ECM for bone cells adhesion, despite the sample-to-sample variability inherent with the choice of such a biomimetic material, and FSS is considered as an externally controllable stimulus able to promote the tissue growth rate.

In the first part, the flow resistance of each sample is measured by means of perfusion tests under two temperature levels, and a pore size distribution characterization is carried out using optical acquisitions on both sides of each sample.

In the second part, the issue of measuring the effective shear stress and its distribution inside the mineral matrix is addressed by creating 3D models of three samples, characterized by largely varying flow resistance, as measured in the first part of the study. The visual characterization is used to replicate the same sample-specific channel size distribution, with the aim to reproduce the same degree of variability among specimens. Simulation results are at first compared against the measured flow resistance with good agreement, and flow velocity profiles are validated against the analytical solution for developed flows in laminar cylinder channels. Then, simulations results are used to study the FSS in the inner channels of the scaffold, revealing that:

- Close to the channels inlet, a higher shear stress is present due to the developing flow velocity profile. This is relevant for bone cells deposited in this area, as they will experience a higher-than-expected shear stress.
- Cumulative probability functions are used to derive exponential expressions for the 10%, 50% and 90% of the shear stress distribution acting on the whole inner solid–fluid interface, as a function of the flow resistance. This relevantly allows to accept the sample-to-sample variability of biomimetic B-HA, which can be characterized with a simple flow resistance test. Based on this, low-order expressions can be used to infer the amount of the shear stress percentiles. Planned developments of the study will aim to obtain the same information from optical acquisition analysis, thus via a non-intrusive technique.

The study combines both experimental and numerical engineering with the research for innovative biological materials, and the use of different investigation approaches (experiments and 3D-CFD simulations) improves the understanding of the flow-biological interaction in 3D scaffolds for BTE.
